# COVID-19 Myocarditis Presenting With New-Onset Heart Failure With Mildly Reduced Ejection Fraction and Rare Ventricular Quadrigeminy

**DOI:** 10.7759/cureus.111954

**Published:** 2026-07-02

**Authors:** Anthony Costa, Sydney Moriarty, John P Martinez, Garrett Beck, Thomas Vanhecke

**Affiliations:** 1 Internal Medicine, Henry Ford Genesys, Grand Blanc, USA; 2 Internal Medicine, Henry Ford Health System, Grand Blanc, USA; 3 Cardiology, Henry Ford Genesys, Grand Blanc, USA

**Keywords:** cardiac inflammation, covid 19, covid and myocarditis, heart failure patients, myocardial speckling, quadrigeminy, sars-cov-2, ventricular arrhythmias

## Abstract

COVID-19 is linked to diverse cardiovascular complications, including myocarditis, new-onset heart failure, and ventricular arrhythmias. Although premature ventricular contractions and bigeminy are recognized, the specific pattern of quadrigeminy remains rarely documented in this setting.

A 57-year-old male with untreated hypertension presented with acute exertional chest pressure, dyspnea, and nocturnal wheezing over two weeks. On arrival, BP was 176/100 mmHg, HR was 101 bpm, and O2 saturation was 93% on room air. ECG showed sinus tachycardia with bigeminy and trigeminy, without QT or QTc prolongation. Lab results showed troponin 0.04 ng/mL, B-type natriuretic peptide (BNP) 241 pg/mL, and the severe acute respiratory syndrome coronavirus 2 (SARS-CoV-2) polymerase chain reaction (PCR) was positive. Chest CT revealed pulmonary edema, and echocardiogram showed a left ventricular ejection fraction (LVEF) of 40-45% with myocardial speckling. Telemetry showed runs of bigeminy, trigeminy, and quadrigeminy. He was diagnosed with acute hypoxic respiratory failure and COVID-19 myocarditis complicated by heart failure with mildly reduced ejection fraction (HFmrEF) and ventricular arrhythmias. Long-term follow-up is ongoing.

This single-case report is based on comprehensive clinical, laboratory, electrocardiographic, telemetry, and imaging data from a community hospital admission. However, the limitations of this report are that this was a single case without cardiac MRI or biopsy confirmation. Hence, causality cannot be definitively established.

To conclude, COVID-19 infection can cause myocarditis complicated by HFmrEF with rare arrhythmias, including quadrigeminy. Careful evaluation and individualized treatment are critical for optimal outcomes.

## Introduction

Severe acute respiratory syndrome coronavirus 2 (SARS-CoV-2) infection remains a significant clinical concern even years into the pandemic. Although most cases of COVID-19 are mild, the virus exhibits clear cardiotoxic potential through direct viral invasion, immune-mediated inflammation, microvascular dysfunction, and cytokine storm [1]. We report a case of COVID-19-associated myocarditis presenting with new-onset heart failure with mildly reduced ejection fraction (HFmrEF) and the uncommon ventricular arrhythmia pattern of quadrigeminy. Myocarditis associated with COVID-19 is well-documented, with incidence estimates varying widely but carrying substantial morbidity [2]. COVID-19-associated myocardial injury occurs in 7% to 40% of hospitalized patients, while clinically suspected myocarditis is less common but carries an adverse prognosis. Ventricular arrhythmias, ranging from premature ventricular contractions to sustained ventricular tachycardia, occur in approximately 2.5% to 5% of COVID-19 patients and are independently associated with increased mortality [3,4]. However, repetitive ventricular quadrigeminy in the setting of COVID-19 myocarditis has rarely been described, and its clinical significance remains poorly defined. Acute viral myocarditis often presents during the inflammatory phase with polymorphic and irregular ventricular ectopy (e.g., bigeminy, trigeminy, quadrigeminy) due to myocardial irritability and heterogeneous repolarization. In contrast, healed or chronic phases more commonly feature monomorphic reentrant arrhythmias around scar tissue. Ventricular quadrigeminy is particularly uncommon and serves as a marker of evolving myocardial inflammation [5,6]. We report a case of COVID-19-associated myocarditis presenting with new-onset HFmrEF (left ventricular ejection fraction (LVEF) of 40-45%) and progression to sustained periods of ventricular quadrigeminy, highlighting the value of telemetry monitoring and supportive multidisciplinary care.

## Case presentation

A 57-year-old Caucasian male with a history of untreated hypertension and class I obesity presented to the emergency department for shortness of breath. He described two weeks of progressive central chest pressure (heaviness, 3/10 intensity), exertional dyspnea, generalized weakness, and nocturnal wheezing that worsened when lying flat. Symptoms began after heavy lifting and were not associated with fevers, productive cough, palpitations, syncope, or peripheral edema. On arrival to ED, blood pressure was 176/100 mmHg, heart rate (HR) 101 bpm, respiratory rate (RR) 18 breaths/min, and oxygen saturation 93% on room air (improving to 95% on a 2 L nasal cannula). Physical examination showed no jugular venous distention or pulmonary crackles, but asymmetric right lower extremity edema was present. The initial electrocardiogram demonstrated sinus tachycardia with frequent ventricular bigeminy without ischemic changes or QT prolongation (Figure 1). Admission laboratory evaluation revealed high-sensitivity troponin peaking at 0.04 ng/mL, B-type natriuretic peptide (BNP) 241 pg/mL, D-dimer 652 ng/mL, and lymphocytosis (Table 1). SARS-CoV-2 PCR was positive.

**Figure 1:**
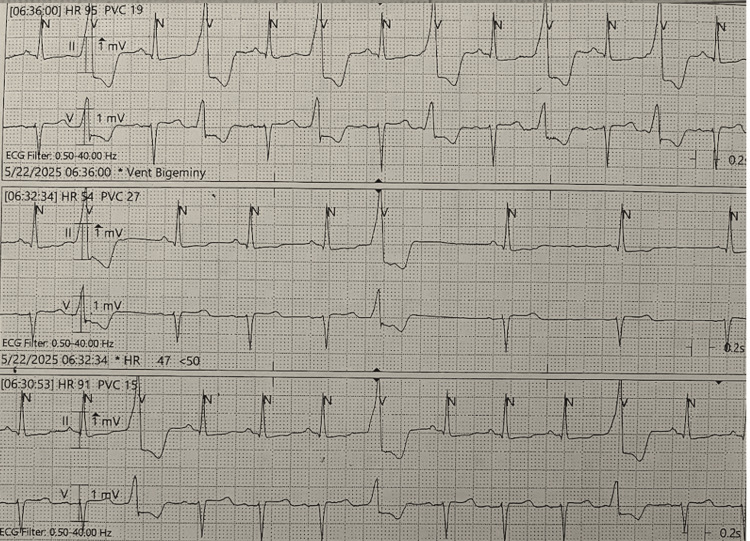
Telemetry monitoring showed frequent premature ventricular contractions that evolved into sustained periods of bigeminy, trigeminy, and multiple runs of quadrigeminy

**Table 1:** Key laboratory values on admission

Laboratory Test	Patient Value	Normal Reference Range	Units	Interpretation/Notes
High-Sensitivity Troponin (peak)	0.04	<0.034 (or lab-specific 99th percentile)	ng/mL	Mild elevation; consistent with myocardial injury/inflammation
BNP	241	<100	pg/mL	Mildly elevated; supports heart strain/edema
D-dimer	652	<500	ng/mL	Mildly elevated; non-specific (COVID, inflammation)
White Blood Cell Count	(Lymphocytosis noted)	4.5–11.0	×10³/µL	Lymphocytosis; viral pattern (COVID)
SARS-CoV-2 PCR	Positive	Negative	N/A	Confirmed infection

Chest CT angiography ruled out pulmonary embolism but showed diffuse interlobular septal thickening, small bilateral pleural effusions, and ground-glass opacities consistent with pulmonary edema and possible COVID-19 pneumonia (Figure 2). Transthoracic echocardiogram demonstrated an LVEF of 40-45%, global hypokinesis, and increased myocardial echogenicity (“speckling”) suggestive of myocardial inflammation (Figure 3; arrows pointing to the speckled myocardium and hypokinetic segments). Continuous telemetry monitoring showed frequent premature ventricular contractions, which evolved into sustained periods of bigeminy, trigeminy, and multiple runs of quadrigeminy (Figure 4). No sustained ventricular tachycardia occurred. Management was multidisciplinary and supportive. Cardiology recommended continued telemetry, serial troponins, and echocardiography. Pulmonology advised dexamethasone for COVID-19 and evaluation for underlying obstructive sleep apnea. The patient received intravenous furosemide 40 mg daily with transition to oral, metoprolol succinate 100 mg daily for HR control and arrhythmia suppression, dexamethasone 6 mg daily for 10 days, and a full course of nirmatrelvir/ritonavir. Aspirin 81 mg daily was added. By hospital day 4 (May 24, 2025), the patient’s chest pressure, dyspnea, and hypoxia had completely resolved. The arrhythmia burden decreased significantly to infrequent premature ventricular contractions (PVCs) without quadrigeminy. He was discharged on metoprolol succinate 100 mg daily, furosemide 40 mg daily, and aspirin 81 mg daily, with close follow-up arranged with cardiology, pulmonology, and primary care.

**Figure 2:**
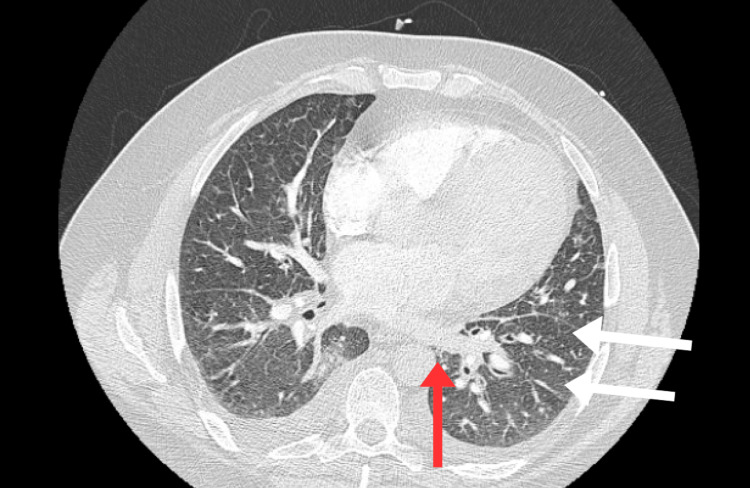
CTA chest (axial) The red arrow points to an area with no pulmonary emboli. The white arrows point to ground-glass opacities and pulmonary edema.

**Figure 3:**
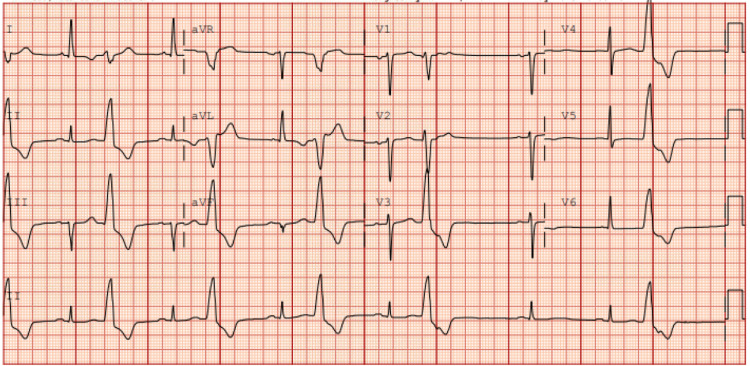
Ventricular bigeminy HR 89, QT 423, and QTc 515

**Figure 4:**
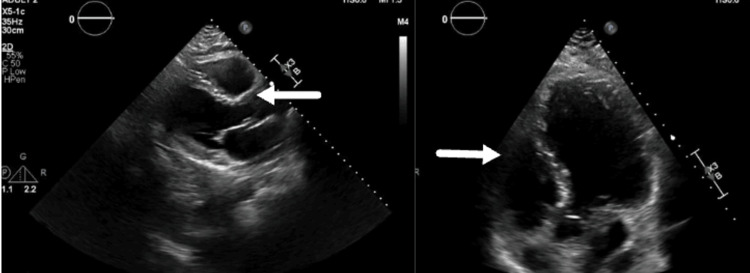
Echocardiogram showing myocardial speckling concerning for myocarditis (white arrows)

Table 2 lists the clinical timeline of this case.

**Table 2:** Clinical timeline BNP: B-type natriuretic peptide; TTE: transthoracic echocardiogram; PVCs: premature ventricular contractions; VT: ventricular tachycardia

Time Point	Key Clinical Events	Findings / Interventions
~2 weeks prior to admission	Heavy lifting followed by symptom onset	Progressive central chest pressure (3/10), exertional dyspnea, generalized weakness, nocturnal wheezing/orthopnea. No fever, cough, palpitations, or edema.
Day of admission (ED)	Presentation to the Emergency Department	BP 176/100 mmHg, HR 101 bpm, RR 18, SpO₂ 93% → 95% on 2L NC. ECG: sinus tachycardia with frequent ventricular bigeminy (Figure 1). Labs: hs-Troponin peak 0.04 ng/mL, BNP 241 pg/mL, D-dimer 652 ng/mL, lymphocytosis, SARS-CoV-2 PCR positive (Table 1). Chest CTA: diffuse interlobular septal thickening, small bilateral pleural effusions, ground-glass opacities (Figure 4). TTE: LVEF 40–45%, global hypokinesis, speckled myocardium (Figure 3).
Hospital Days 1–3	Inpatient monitoring and treatment	Telemetry: frequent PVCs evolving to bigeminy, trigeminy, and runs of quadrigeminy (Figure 2). No sustained VT. Treatment: IV furosemide 40 mg daily (then oral), metoprolol succinate 100 mg daily, dexamethasone 6 mg daily ×10 days, nirmatrelvir/ritonavir, aspirin 81 mg daily.
Hospital Day 4 (May 24, 2025)	Clinical improvement and discharge	Complete resolution of chest pressure, dyspnea, and hypoxia. Arrhythmia burden markedly decreased to infrequent PVCs. Discharged on metoprolol succinate 100 mg daily, furosemide 40 mg daily, and aspirin 81 mg daily. Follow-up arranged with Cardiology, Pulmonology, and Primary Care.

## Discussion

Viral infections, such as COVID-19, are a common precipitant of myocarditis [1,4]. Acute myocarditis may cause arrhythmias, heart failure, cardiogenic shock, and thromboembolic events [2,3]. During the inflammatory phase of myocarditis, polymorphic and irregular arrhythmias are more common, whereas healed myocarditis more often produces monomorphic arrhythmias [2]. During the acute inflammatory phase of myocarditis, polymorphic and irregular ventricular ectopy, such as bigeminy, trigeminy, and quadrigeminy, predominate due to myocardial irritability and heterogeneous repolarization. In contrast, healed or chronic myocarditis more commonly produces monomorphic ventricular tachycardia from reentrant circuits around scar tissue [2]. This case is notable for the relatively rare finding of ventricular quadrigeminy in the setting of COVID-19 myocarditis with new-onset HFmrEF. The progression from frequent premature ventricular contractions to bigeminy, trigeminy, and finally quadrigeminy on telemetry provided an important clinical clue to evolving myocardial inflammation, even before marked troponin elevation or severe systolic dysfunction became apparent. Myocardial speckling on echocardiography further supported an inflammatory process [4]. Such atypical arrhythmia patterns, while infrequently highlighted in the literature, likely reflect the intense, patchy myocardial inflammation characteristic of acute viral myocarditis [2]. This case emphasizes the importance of vigilant rhythm monitoring, as subtle telemetry findings may represent evolving myocarditis. With no standardized management guidelines [4-6], supportive therapy remains the cornerstone of treatment [2]. In the absence of standardized, evidence-based management guidelines specific to COVID-19 myocarditis [1,4], treatment remains largely supportive and individualized. Our patient responded rapidly to a multimodal regimen consisting of diuresis with furosemide for volume overload, beta-blockade with metoprolol succinate for heart rate control and arrhythmia suppression, corticosteroids (dexamethasone), and an antiviral agent (nirmatrelvir/ritonavir). This approach aligns with expert consensus recommendations for acute COVID-19 cardiovascular syndrome [1,7-9] and resulted in complete symptom resolution and marked reduction in arrhythmia burden within four days.

Several limitations must be acknowledged. Cardiac magnetic resonance imaging and endomyocardial biopsy were not performed, limiting definitive confirmation of myocarditis. Long-term follow-up data, including repeat echocardiography and arrhythmia monitoring, are pending. While the clinical picture strongly suggests COVID-19 as the trigger, coincidental etiologies cannot be entirely excluded.

Differential diagnosis

Although clinically suspected COVID-19-associated myocarditis was considered the most likely diagnosis, alternative etiologies for the patient's reduced left ventricular ejection fraction and ventricular ectopy were also considered. Untreated long-standing hypertension may contribute to hypertensive cardiomyopathy and mild systolic dysfunction, while occult ischemic heart disease remains an important cause of newly reduced ejection fraction in middle-aged adults. However, several features favored myocarditis. The patient developed symptoms during acute SARS-CoV-2 infection, had mild troponin elevation without ischemic electrocardiographic changes, demonstrated global rather than regional left ventricular hypokinesis, and exhibited progressive ventricular ectopy that improved with supportive treatment. Although coronary angiography, cardiac magnetic resonance imaging, and endomyocardial biopsy were not performed, the overall clinical presentation was felt to be most consistent with clinically suspected COVID-19-associated myocarditis. Nevertheless, ischemic and hypertensive cardiomyopathy cannot be completely excluded and represent important limitations of this report.

## Conclusions

COVID-19 infection can precipitate myocarditis complicated by HFmrEF and rare arrhythmias such as quadrigeminy. Careful clinical evaluation, including serial telemetry and echocardiography with attention to subtle findings (e.g., arrhythmia progression and myocardial speckling), combined with individualized supportive management (diuresis, beta-blockade, steroids, and antivirals), can yield favorable short-term outcomes. This case reinforces the cardiotoxic potential of SARS-CoV-2 and the value of multidisciplinary care with longitudinal surveillance.
